# Prognostic role of CD4 T-cell depletion after frontline fludarabine, cyclophosphamide and rituximab in chronic lymphocytic leukaemia

**DOI:** 10.1186/s12885-019-5971-z

**Published:** 2019-08-14

**Authors:** Martin Gauthier, Françoise Durrieu, Elodie Martin, Michael Peres, François Vergez, Thomas Filleron, Lucie Obéric, Fontanet Bijou, Anne Quillet Mary, Loic Ysebaert

**Affiliations:** 1 Department of Haematology, Toulouse-Oncopole University Cancer Institute (IUCT-O), 1 Avenue Irene Joliot-Curie, 31059 Toulouse, France; 2 Department of Biology Haematology, Institut Bergonié, Bordeaux, France; 3 Department of Biostatistics, Institut Claudius Regaud, IU, CT-O Toulouse, France; 4 Department of Biology Haematology, Toulouse-Oncopole University Cancer Institute (IUCT-O), Toulouse, France; 5 Department of Medical Haematology, Institut Bergonié, Bordeaux, France; 6 Inserm UMR1037, Cancer Research Centre of Toulouse, Toulouse, France

**Keywords:** Chronic lymphocytic Leukaemia, Minimal residual disease, CD4 T-cells, Immunosuppression, Chemo-immunotherapy

## Abstract

**Background:**

Eradication of minimal residual disease (MRD), at the end of Fludarabine-Cyclophosphamide-Rituximab (FCR) treatment, is a validated surrogate marker for progression-free and overall survival in chronic lymphocytic leukaemia. But such deep responses are also associated with severe immuno-depletion, leading to infections and the development of secondary cancers.

**Methods:**

We assessed, blood MRD and normal immune cell levels at the end of treatment, in 162 first-line FCR patients, and analysed survival and adverse event.

**Results:**

Multivariate Landmark analysis 3 months after FCR completion identified unmutated *IGHV* status (HR, 2.03, *p* = 0.043), the level of MRD reached (intermediate versus low, HR, 2.43, *p* = 0.002; high versus low, HR, 4.56, *p* = 0.002) and CD4 > 200/mm^3^ (HR, 3.30, *p* <  0.001) as factors independently associated with progression-free survival (PFS); neither CD8 nor NK counts were associated with PFS. The CD4 count was associated with PFS irrespective of *IGHV* mutational status, but only in patients with detectable MRD (HR, 3.51, *p* = 0.0004, whereas it had no prognostic impact in MRD < 10^− 4^ patients: *p* = 0.6998). We next used a competitive risk model to investigate whether immune cell subsets could be associated with the risk of infection and found no association between CD4, CD8 and NK cells and infection.

**Conclusions:**

Consolidation/maintenance trials based on detectable MRD after FCR should investigate CD4 T-cell numbers both as a selection and a response criterion, and consolidation treatments should target B-cell/T-cell interactions.

## Background

In chronic lymphocytic leukaemia (CLL), chemo-immunotherapy (CIT) with fludarabine, cyclophosphamide and rituximab (FCR) is now well established as a standard of care for young treatment-naive, fit patients without *TP53* locus alterations (mutations and/or deletions) and with normal renal function [1, 2]. When compared to new generation targeted signalling inhibitors, FCR induces very prolonged remission periods in a subset of patients with *IGHV* mutations (*IGHV-M*), with three independent long-term follow-up studies reporting a > 10 year progression-free survival (PFS), specifically in patients in whom minimal residual (MRD) cannot be detected (< 10^− 4^) after treatment completion [3–5]. In a pooled analysis from randomised trials, FCR treatment of patients without *IGHV* mutations (*IGHV-UM*) resulted in a median PFS of only 42.9 months, with the absence of a plateau on the PFS curve and an attenuation of the advantages of reaching an undetectable MRD status [6]. In the context of CIT, the evaluation of MRD is of utmost importance because patients with undetectable MRD after treatment still achieve better PFS and overall survival (OS) than those with detectable MRD [7–12]. The quantification of MRD is however not recommended beyond the context of clinical trials [13, 14].

A number of factors are known to be associated with the depth of MRD response achieved by CIT (*TP53* mutation and/or deletion 17p [del17p], high β2-microglobulin levels, or complex karyotype). Conversely, we have a limited understanding of the factors that influence an almost universal relapse in *IGHV-UM* patients, despite achieving undetectable MRD status [6]. Indeed, there is a lack of clinical factors that can accurately improve the prognostic power of eradicating MRD [15]. Since bystander immune cells such as CD4 T-cells promote CLL survival/proliferation in tumour niches *before* FCR [16], we hypothesised that normal lymphocyte levels may influence the duration of PFS independently of the MRD status achieved *after* completion of therapy. Since FCR also induces profound and durable lymphopenia, we correlated these measurements to the well-described risk of developing secondary malignancies and/or serious infectious events [17].

## Methods

### Study population

Between January 01, 2005 and February 29, 2016, 162 patients receiving frontline FCR for CLL in two institutions (IUCT-Oncopôle, Toulouse and Institut Bergonié, Bordeaux, France) were enrolled in our study. Patients’ clinical and biological data were retrieved from medical charts. In addition to complete blood counts, flow cytometry analyses were performed on peripheral blood samples at the end of treatment (EOT, i.e. 3 months after the last course of FCR) to monitor both normal immune reconstitution (CD4, CD8, NK) and MRD levels. MRD was quantified by 8-colour flow cytometry, with a sensitivity of at least 10^− 4^, using a combination (MRD antibody cocktail) comprising CD81-FITC (BD Pharmingen), CD43-PE (Beckman Coulter), CD79b-PerCP Cy5.5 (BD Biosciences), CD5-PC7 (Beckman Coulter), CD22-APC (BD Biosciences), CD20-AA700 (Biolegend), CD45-APC-H7 (BD Biosciences) and CD19-BV510 (BD Biosciences). One to five hundred microliters of fresh blood were incubated with the MRD antibody cocktail for 15 min, then red cells were lysed (with BD lysis buffer) for 15 min and washed twice. Flow cytometry analysis of a minimum of 10^5^ leucocytes was carried out on a Navios instrument with Kaluza software (Beckman Coulter). Residual CLL cell gating and quantification was assessed according to the ERIC recommendations [18–20].

### Definition of outcomes

Progression-free survival (PFS) was calculated from the first day of the first cycle of FCR (D1C1) to either relapse (per IwCLL2008 recommendations) or death, from any cause [13]. Overall survival (OS) was calculated from D1C1 FCR to death, from any cause. At the end of treatment (EOT, i.e. 3 months after the last course of FCR), the overall response rate was classed as either complete clinical response (clinical CR), complete response with incomplete bone marrow recovery (CRi), partial response (PR), or failure. This response assessment differed from the IwCLL2008 criteria, in that bone marrow biopsies are not warranted beyond the context of clinical trials in France; this explains why we used the term “clinical CR” instead of complete response (CR). MRD levels were classified as undetectable (< 10^− 4^), intermediate (10^− 4^ to 10^− 2^) and high (≥10^− 2^), as defined by the German CLL study group in the CLL8 and CLL10 trials [7, 21].

Opportunistic infections were described as follows: herpes zoster, *Pneumocystis* pneumonia, CMV disease, infection-driven hemophagocytic lymphohistiocytosis, invasive fungal infection, *Toxoplasma gondii* infection, malignant external otitis, progressive multifocal leukoencephalopathy, hepatitis B re-activation (in patients who were previously both anti-hepatitis B core and anti-hepatitis B surface antigen positive), and chronic hepatitis E infection. Severe infections were defined as any infection leading to hospitalisation (irrespectively of a common terminology criteria grade). Patients received primary prophylaxis with trimethoprim-sulfamethoxazole and valaciclovir in > 90% of cases (stopped 6 months after EOT evaluation in most cases [22]).

### Statistical analyses

Continuous variables were presented as the median with a range (min-max) and categorical variables were summarised by frequencies and percentages. EOT CD4 counts were evaluated as a binary covariable with a threshold of 200/mm^3^, typically used to guide infection prophylaxis in HIV patients [23], but also in routine haematology practice. NK, CD8 and monocyte count cut-offs used were based on the median count at EOT.

The chi-square or Fisher’s exact test was used to compare categorical variables. Survival rates were estimated by Kaplan-Meier, with 95% confidence intervals (95%CI). Patients that were still alive were censored at the cut-off date or at their last available follow-up. Univariate and multivariate analyses were performed using the Logrank test and the Cox proportional hazards model; Hazard Ratios (HR) were estimated with 95% confidence intervals. Landmark analyses were performed at 9 months after initiation of treatment, to assess the impact of variables evaluated post-treatment on OS and PFS. Cumulative incidences of opportunistic and/or serious infections were estimated using a competing risks model, with relapse and death considered as competing events. Univariate analyses were performed using the Fine and Gray model and sub Hazard Ratios were estimated with a 95%CI. All tests were two-sided and *p* values < 0.05 were considered statistically significant. All analyses were conducted with STATA v13 (Stata Corporation, College Station, TX, USA) and R (3.4.3).

## Results

### Pre-therapy cohort characteristics

Patients’ characteristics are summarised in Table 1. Patients were males in 69.1% of cases. The median age was 61.5 years and Binet stage was B/C in 79% of patients. Other known prognostic variables included: 11q deletion in 22.2%, 17p deletion in 3.9%, *IGHV-UM* status in 63.4%, β2-microglobulin > 3.5 mg/L in 78.3%, complex karyotype in 21.6%, *NOTCH1* mutations in 16.2%, and *SF3B1* mutations in 8.8% of patients. The majority (75.9%) of patients received 6 cycles of FCR, and 98.1% received at least 4 cycles.

**Table 1:** Patients’ pre-treatment characteristics

Pre-FCR characteristics	n (%) or median [min-max]
Gender	
•Men	112 (69.1)
•Women	50 (30.9)
Age category	
•>65 years	58 (35.8)
•≤65 years	104 (64.2)
Binet stage	
•Binet A	34 (21.3)
•Binet B	85 (53.1)
•Binet C	41 (25.6)
•Missing	2
Time from diagnosis to FCR (months)	22.1 [0.03-203.00]
Lymphocytes (G/L) (n=114)	90.6 [1.30-888.0]
β2-microglobulin	
•≤3.5 mg/L	15 (21.7)
•>3.5 mg/L	54 (78.3)
•Missing	93
LDH value	
•LDH≤ULN	52 (45.6)
•LDH>ULN	62 (54.4)
•Missing	48
*IGHV* mutation status	
•*IGHV-M*	48 (36.6)
•*IGHV-UM*	83 (63.4)
•Missing	31
Del13q	
•Del13q	49 (37.4)
•No del13q	82 (62.6)
•Missing	31
Trisomy 12	
•Trisomy 12	32 (24.4)
•No trisomy 12	99 (75.6)
•Missing	31
Del11q	
•Del11q	34 (22.2)
•No del11q	119 (77.8)
•Missing	9
Del6q	
•Del6q	14 (12.8)
•No del6q	95 (87.2)
•Missing	53
t(14;18)(q32;q21)	
•t(14;18)(q32;q21)	5 (4.6)
•No t(14;18)(q32;q21)	104 (95.4)
•Missing	53
Del17p	
•Del17p	6 (3.9)
•No del17p	146 (96.1)
•Missing	10
Complex karyotype	
•Complex karyotype	87 (78.4)
•No complex karyotype	24 (21.6)
•Missing	51
*TP53* mutation	
•*TP53* mutation	2 (2.9)
•No *TP53* mutation	68 (97.1)
•Missing	92
*NOTCH1* mutation	
•*NOTCH1* mutation	11 (16.2)
•No *NOTCH1* mutation	57 (83.8)
•Missing	94
*SF3B1* mutation	
•*SF3B1* mutation	6 (8.8)
•No *SF3B1* mutation	62 (91.2)
•Missing	94
*MYD88* mutation	
•*MYD88* mutation	1 (1.5)
•No *MYD88* mutation	66 (98.5)
•Missing	95
*BRAF* mutation	
•No *BRAF* mutation	67 (100)
•Missing	85

### Response rates, PFS and MRD assessment

**Fig. 1:**
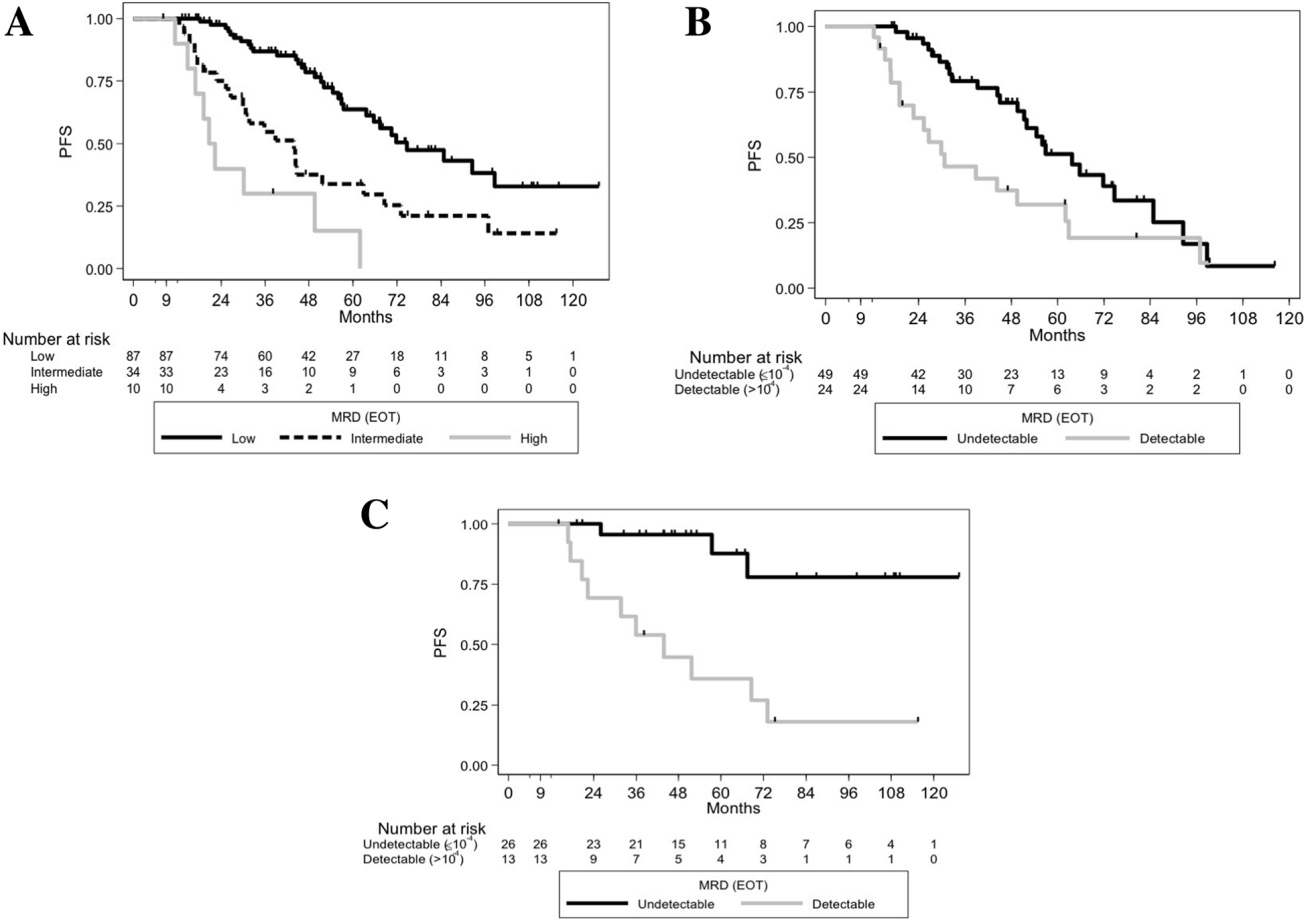
PFS of the different EOT MRD level groups, and according to *IGHV* mutational status. (A) PFS of the different MRD level groups at EOT in the whole population (both *p* < 0.0001 for low versus intermediate and low versus high levels). (B) PFS according to EOT MRD status (detectable versus undetectable) in *IGHV*-unmutated patients (*p* = 0.0206). (C) PFS according to EOT MRD status (detectable versus undetectable) in *IGHV*-mutated patients (*p* = 0.0002). EOT: end of treatment, MRD: minimal residual disease

The overall response rate was 98.8%, with 96.2% of patients achieving clinical CR/CRi. An EOT MRD assessment was available for 147 patients, of these 65.3% achieved undetectable MRD, 27.2% achieved intermediate levels, and 7.5% had high levels. After a median follow-up (FU) of 60.5 months (95%CI [54.0–71.5]), 46.3% of patients relapsed or died, with a median PFS of 65.7 months (95%CI [54.5–74.7]). In the univariate Cox model, baseline characteristics associated with shorter PFS were *IGHV-UM* (HR, 2.55 [1.42–4.59], *p* = 0.0012), del17p and/or *TP53* mutation (HR, 3.87 [1.34–11.22], *p* = 0.0072) and del11q (HR, 2.19 [1.35–3.56], *p* = 0.0012) (Table 2). As expected from previous studies, EOT MRD levels were associated with PFS (intermediate versus low HR, 2.64 [1.55–4.50] *p* = 0.0004, high versus low HR, 6.95 [3.24–14.92], *p* <  0.0001) (Fig. 1A). With regards to *IGHV* mutational status, in *IGHV-M* patients, the 5-year PFS rate was 87.7% in MRD undetectable versus 35.9% in MRD detectable, against 51.2% versus 31.9% in *IGHV-UM* patients respectively (Fig. 1B-1C). Notably, patients with detectable MRD levels had comparable 5-year PFS rates irrespective of their *IGHV* mutational status.

**Table 2:** Factors associated with progression-free survival (PFS) by univariate and multivariate analysis

PFS	univariate analysis		multivariate analysis	
Variables	HR [95%CI]	p	HR [95%CI]	p
11q deletion (*n* = 153)				
No (ref)	1.00		1.00	
Yes	2.19 [1.35; 3.56]	**0.0012**	1.74 [0.94; 3.21]	0.076
*IGHV* mutation status (*n* = 131)				
Mutated (ref)	1.00		1.00	
Unmutated	2.55 [1.42; 4.59]	**0.0012**	2.03 [1.02; 4.04]	**0.043**
EOT MRD^a^ (*n* = 131)				
Low (ref)	1.00		1.00	
Intermediate	2.64 [1.55; 4.50]		2.43 [1.39; 4.27]	**0.002**
High	6.95 [3.24; 14.92]	**< 0.0001**	4.56 [1.76;11.79]	**0.002**
EOT CD4^a^ (/mm^3^, *n* = 132)				
≤ 200 (ref)	1.00		1.00	
> 200	2.28 [1.35; 3.86]	**0.0016**	3.30 [1.79; 6.06]	**< 0.001**
EOT NK^a^ (/mm^3^, *n* = 109)				
≤ 100 (ref)	1.00		–	–
> 100	1.03 [0.60; 1.78]	0.9101		
EOT CD8^a^ (/mm^3^, *n* = 132)				
≤ 150 (ref)	1.00		–	–
> 150	1.02 [0.62; 1.66]	0.9418		
EOT monocytes^a^ (/mm^3^, *n* = 114)				
≤ 400 (ref)	1.00		–	–
> 400	1.30 [0.77; 2.19]	0.3257		

^a^indicates Landmark analysis at 9 months

### Normal immune cells subsets and PFS

**Fig. 2:**
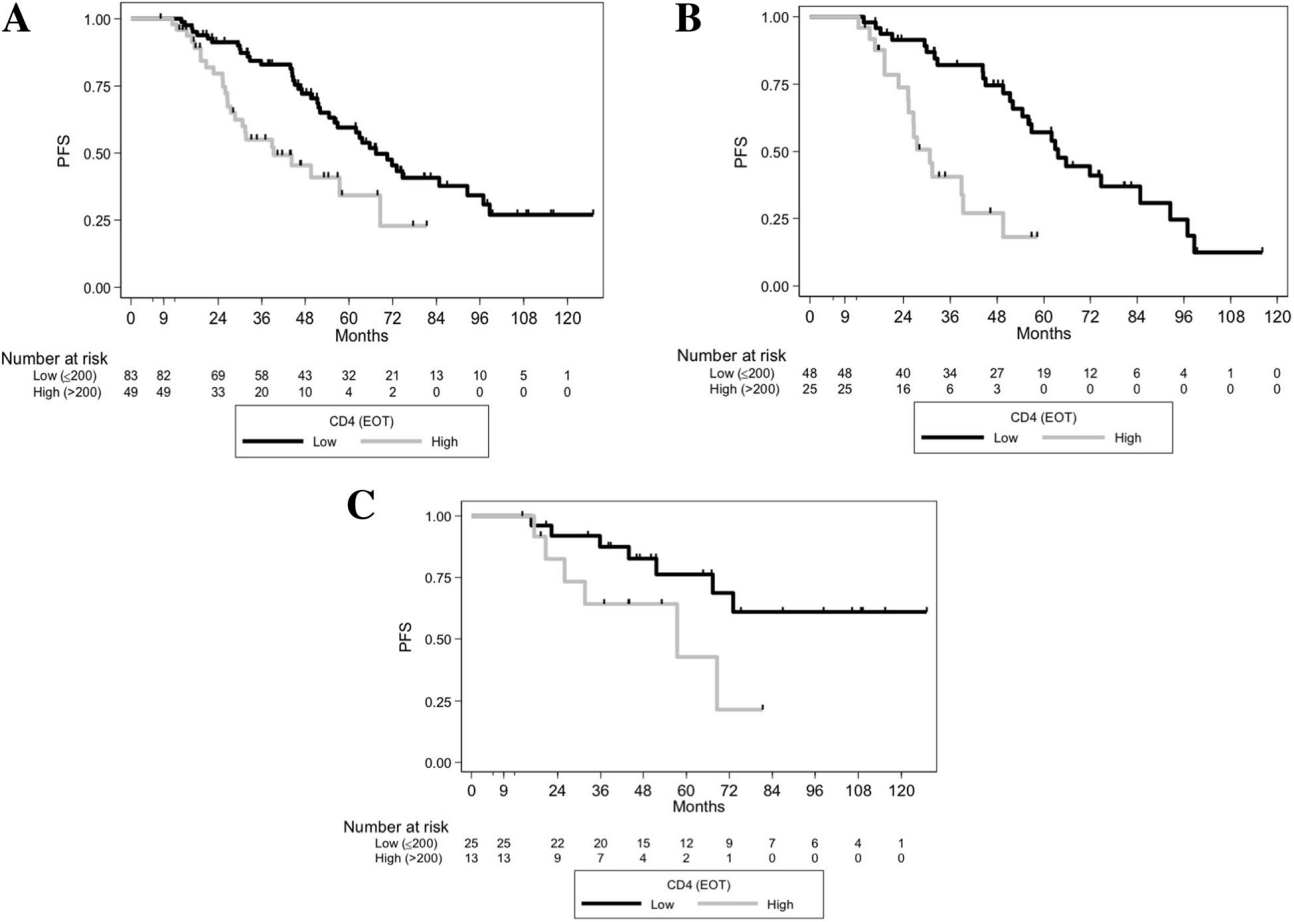
PFS of the different EOT CD4 levels and according to *IGHV* mutational status in the whole population. (A) PFS curves according to EOT CD4 status in the whole population (*p* = 0.0016). (B) PFS curves according to EOT CD4 status in patients with *IGHV*-unmutated status (*p* < 0.0001) (C) PFS curves according to EOT CD4 status in patients with mutated *IGHV* (*p* = 0.0576)

At EOT, the median counts of CD4, CD8 T-cells, monocytes and NK lymphocytes were 154, 153, 418 and 114/mm^3^ respectively. A level of CD4 ≤ 200/mm^3^ was observed in 64.2% of patients. In Landmark analyses, EOT CD4 > 200/mm^3^ was associated with an increased risk of relapse (median PFS 39.3 months versus 67.4 months if EOT CD4 ≤ 200/mm^3^, HR, 2.28 [1.35–3.86] *p* = 0.0016) (Fig. 2A)). PFS was not associated with EOT CD8 T-cell levels (≤150/mm3 versus > 150/mm^3^ *p* = 0.9418), nor with EOT monocyte levels (≤400/mm3 versus > 400/mm^3^ *p* = 0.3257), nor with NK cells (≤100/mm3 versus > 100/mm^3^ *p* = 0.9101). Reaching a low EOT CD4 T-cell count was associated with a trend towards a better PFS in *IGHV-M* patients (5-year PFS of 76.2% versus 42.8%, HR, 2.81 [0.92–8.54], *p* = 0.0576), and with a much greater PFS in *IGHV-UM* patients (median PFS of 63.7 versus 30.7 months, HR, 4.09 [2.00–8.39], *p* < 0.0001, Fig. 2B-2C). In multivariate Landmark analysis (Table 2), the following variables were associated with PFS: *IGHV-UM* (HR, 2.03 [1.02–4.04], *p* = 0.043), EOT CD4 > 200/mm^3^ (HR; 3.30 [1.79–6.06], *p* < 0.001) and EOT MRD (intermediate versus low, HR, 2.43 [1.39–4.27], *p* = 0.002; high versus low, HR, 4.56 [1.76–11.79], *p* = 0.002).

### PFS and CD4 counts in different MRD subgroups

**Fig. 3:**
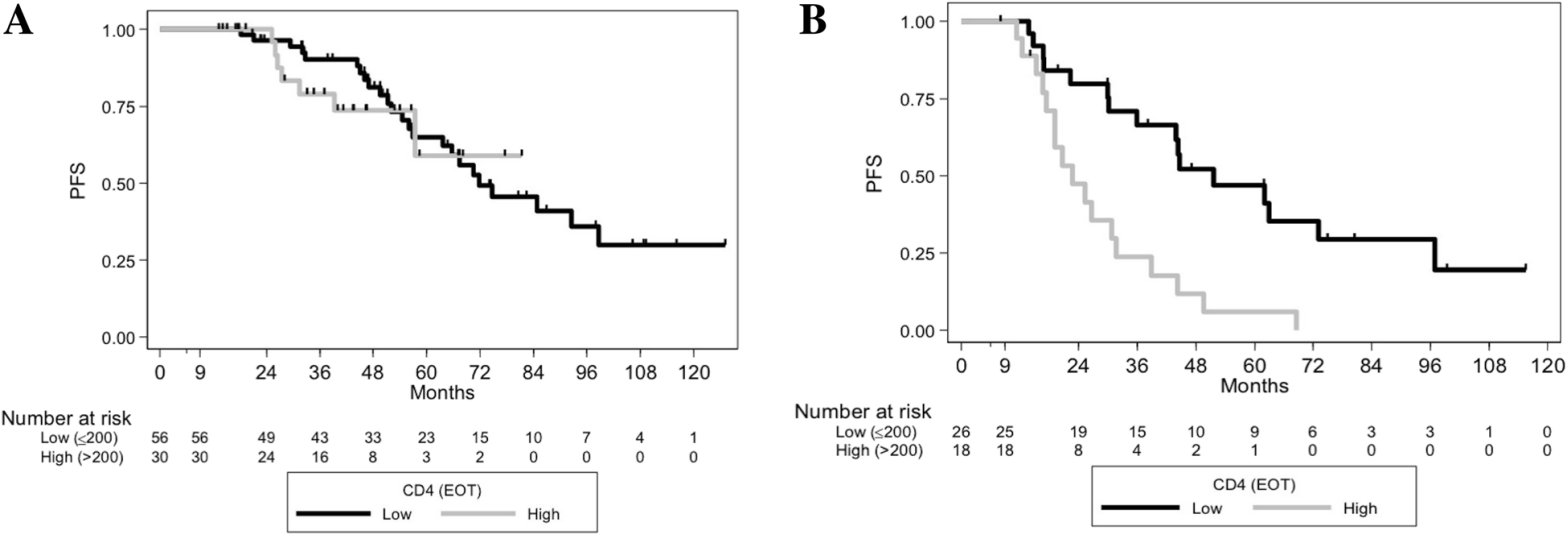
PFS according to EOT CD4, and according to EOT MRD levels. (A) PFS curves according to EOT CD4 status in patients with undetectable (< 10^−4^) EOT MRD (*p* = 0.6998). (B) PFS curves according to EOT CD4 status in patients with detectable (≥10^− 4^) EOT MRD (*p* = 0.0004)

As the CD4 count was found to be an independent parameter which more accurately redefined PFS according to *IGHV* mutational status, we next sought to investigate its association with PFS in the undetectable and detectable MRD subgroups (due to the very small number of patients with high EOT MRD [*n* = 10], we pooled these patients with the intermediate EOT MRD patients). In the low MRD group (*n* = 86), patients with EOT CD4 ≤ 200/mm^3^ had 5-year PFS of 65% versus 59% if CD4 > 200/mm^3^ (*p* = 0.6998, Fig. 3A). Conversely, in cases with detectable MRD levels at EOT (*n* = 44), patients with EOT CD4 ≤ 200/mm^3^ had a 5-year PFS of 47.03% versus 5.93% if EOT CD4 > 200/mm^3^ (HR, 3.51 95%CI [1.68–7.32], *p* = 0.0004) (Fig. 3B). Taken together, these results suggest that the EOT CD4 count may help clinicians to more accurately predict PFS in patients with detectable MRD levels following FCR treatment.

### Overall survival (OS) and toxicities after FCR

**Fig. 4:**
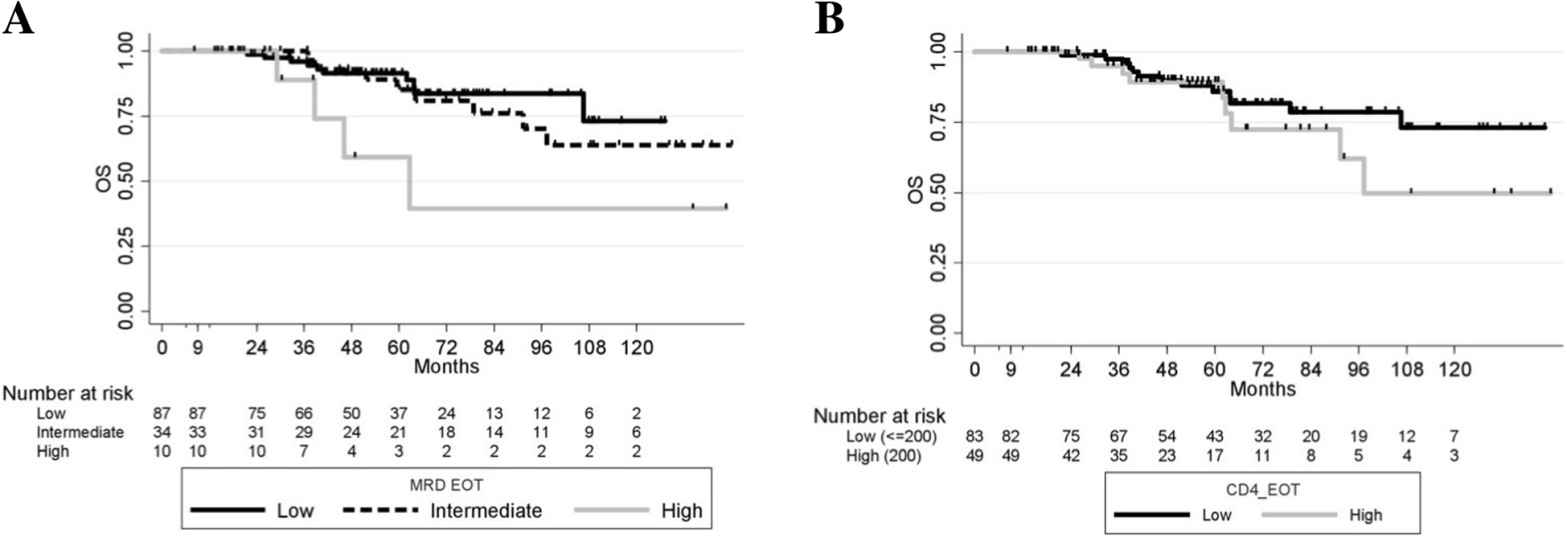
OS according to EOT MRD, and according to EOT CD4 count. (A) OS according to level of EOT MRD (for low versus intermediate, *p* = 0.435 and low versus high levels, *p* = 0.021). (B) OS according to EOT CD4 status in the whole population (*p* = 0.263)

Twenty-five patients (15.4%) died. Five-year OS was 87.7% (95%CI [80.34–92.50]). In Landmark univariate analyses, only a high versus a low level of MRD at EOT was associated with OS (intermediate versus low, HR, 1.45 [0.57–3.72] *p* = 0.435, high versus low, HR, 3.96 [1.23–12.74], *p* = 0.021), whereas the EOT CD4 cell count was not found associated with OS (HR, 1.62 [0.69–3.81], *p* = 0.2631) (Fig. 4). During FU, 20 patients (12.3%) developed a secondary cancer within a median time of 40 months from D1C1 FCR (range, 6–111), and 10 patients (6.2%) developed a Richter transformation (RT) within a median time of 59.5 months from D1C1 FCR (Table 3). Due to the small number of patients with secondary cancers as the first event (*n* = 10), we could not investigate the association of EOT CD4, CD8 and NK cell counts with the incidence of those events; nevertheless, of these 10 patients, 4 had EOT CD4 > 200/mm^3^, 4 had EOT NK > 100/mm^3^ and 5 had EOT CD8 > 150/mm^3^, a proportion rather similar to that measured in the entire cohort.

**Table 3:** Other cancers which developed during follow-up. AML indicates acute myeloid leukaemia.

Cancer	Number
Secondary haematologic	
AML/myelodysplasia	2
Anaplastic large cell lymphoma	1
Multiple myeloma	1
Erdheim-Chester disease	1
Richter transformation	
Diffuse large B-cell lymphoma	9
Hodgkin lymphoma	1
Solid tumours	
Non-melanoma skin cancer	4
Lung cancer	4
Prostate cancer	2
Colorectal cancer	2
Oropharyngeal cancers	2
Breast cancer	1

**Fig. 5:**
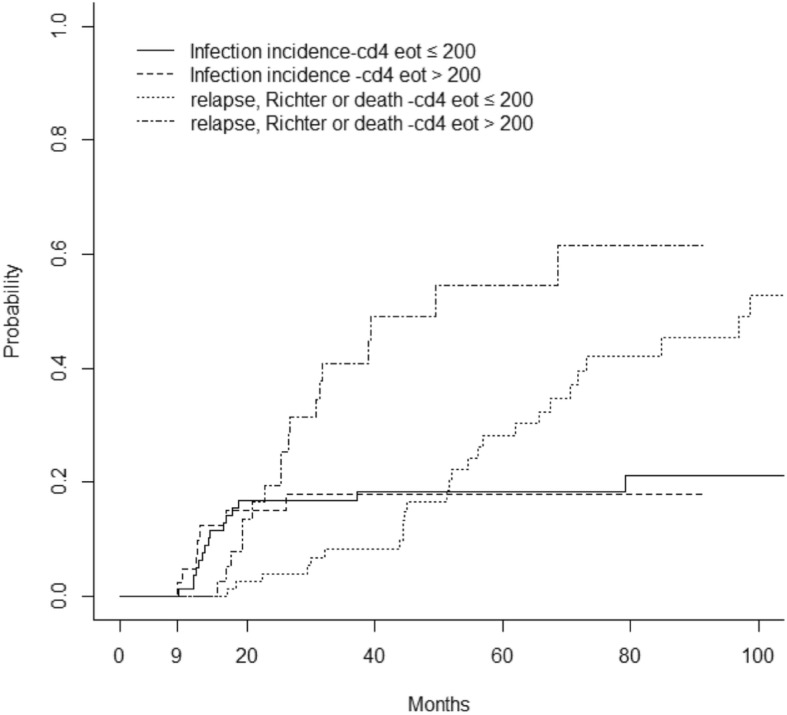
Cumulative incidence of severe and/or opportunistic infections, and of relapse/death according to EOT CD4. Threshold of 200/mm3. High EOT CD4 were associated with higher risk of death/relapse/Richter transformation (HR, 2.34 [1.26–4.33], *p* = 0.007), whereas no association was found between EOT CD4 and severe and/or opportunistic infections (HR, 0.97 [0.40–2.38], *p* = 0.948)

Twenty-five patients developed a serious infection, within a median time of 15 months from D1C1 FCR (range, 2–112), and thirty-five patients developed an opportunistic infection, within a median time of 14 months from D1C1 FCR (range, 2–94). When performing a Landmark analysis at 9 months from D1C1 FCR, the cumulative incidence of serious and/or opportunistic infection was 4.6% at 12 months and 14.9% at 24 months. The competing risk analysis (Table 4) did not detect any association between EOT levels of NK, CD8 or CD4 and serious and/or opportunistic infections. Figure 5 represents the cumulative risk of serious and/or opportunistic infections and relapse or death with the EOT CD4 T-cell count in the entire studied population.

**Table 4:** Univariate analyses of factors associated with severe and/or opportunistic infections. Landmark competing risk analysis at 9 months. sHR indicates sub-Hazard Ratio

	Infections		Relapse/death	
Variables	sHR [95%CI]	p	sHR [95%CI]	p
EOT MRD status (*n* = 125)				
< 10^−4^ (ref)	1.00		1.00	
≥ 10^−4^	0.72 [0.29; 1.82]	0.492	3.02 [1.68; 5.45]	< 0.001
EOT CD4 (/mm^3^, *n* = 125)				
≤ 200 (ref)	1.00		1.00	
> 200	0.97 [0.40; 2.38]	0.948	2.34 [1.26; 4.33]	0.007
EOT NK (/mm^3^, *n* = 119)				
≤ 100 (ref)	1.00		1.00	
> 100	0.51 [0.21; 1.22]	0.128	1.07 [0.58; 1.96]	0.837
EOT CD8 (/mm^3^, *n* = 125)				
≤ 150	1.00		1.00	
> 150	1.84 [0.79; 4.30]	0.158	1.06 [0.59; 1.89]	0.843

Landmark competing risk analysis at 9 months. sHR indicates the sub-Hazard Ratio.

## Discussion

We report results obtained from a large series of patients receiving frontline FCR in the routine practice of two large regions of southwestern France, with a median follow up of over 5 years. Our population was rather similar to that of the CLL8 study and other cohorts, but also included older patients and patients with more advanced disease [1, 4, 21]. We first confirmed the general clinical importance, of achieving a low MRD level at EOT, which extends the relevance of assessing MRD well beyond that of clinical trials. We observed a plateau in the PFS curves of *IGHV-M* patients who achieved a low MRD level endpoint, and also the universal relapse pattern of *IGHV-UM* patients despite eradicating MRD in peripheral blood. In an attempt to better understand this unique feature, we investigated whether normal lymphocyte counts could redefine the prognosis in distinct subgroups of patients. We found that the post-therapy CD4 count was associated with a different prognosis depending on the *IGHV* status, and that this also extended to patients with detectable MRD at EOT. The CD4 count was however not associated with infections, even though this parameter is generally routinely used in clinical practice to determine the start/hold timing of prophylactic measures (with trimethoprim-sulfamethoxazole and/or valaciclovir).

Since no plateau was observed in the PFS curves of low CD4 *IGHV-UM* patients, it is very unlikely that this parameter *alone* could explain the relapse pattern observed in these patients. But in the detectable MRD group, a high CD4 count post-FCR was able to identify a subgroup of patients with a median PFS of only 24 months (a widely accepted definition of FCR-refractory disease [2]). Hence, the CD4 count could help identify patients who may benefit from a consolidation after FCR, especially if the drug modulates T-cells numbers and effects (such as lenalidomide [24–29] or ibrutinib [30–33]). Our previous series was the first to illustrate an effect on CD4 T-cell count following FCR treatment in CLL [34]. A thorough analysis of the phenotype of these T-cells revealed that most were CD4+ CD25+ CD127- FoxP3+ (and as such likely to belong to the T regulatory subset, our unpublished data), which have previously been reported to mediate a CLL-supportive effect in vitro and in vivo [16, 35–37]. Another single-centre retrospective study found that absolute lymphocyte count < 1000/μl three months post-FCR was associated with OS and event-free survival, without MRD data and without analysing the lymphocyte subsets (thus they could not determine the clonal nature of these lymphocytes [38]). In addition to reflecting the pharmacodynamic activity of FCR, we consider lympho-depletion as a more complex, dynamic period of lymphocyte recovery with inter-clonal competitions. It would be surprising that a 3-drug regimen dose effect would be restricted to the CD4 subset (and not to CD8 or NK lymphocytes). Since the prognostic benefits of CD4 T-cells in our study were only observed in patients with detectable residual CLL cells, this argues for a bystander effect rather than just a dose effect. It would be interesting to further investigate CD4 effects in CLL, and to observe whether patients with low EOT CD4 already presented with low CD4 prior to FCR treatment; this could help clinicians identify patients with a high probability of reaching low EOT CD4 after CIT, and thus help select patients who would benefit the most from CIT, which would be a useful distinction to make as FCR is currently being compromised by other first-line therapeutic strategies [39]. Since our research focussed on identifying patients who would benefit from maintenance therapy *after* completing FCR, we did not perform this type of analysis; neither did we perform sequential lymphocyte subset counts during FCR therapy, as has been previously reported in the case of sequential MRD measurements taken during FCR therapy [40].

Furthermore, by highlighting the clinical relevance of CLL cell interactions with their microenvironment in relation to PFS, our research may pave the way for the investigation of associations between other amenable factors (such as CD40 or IL4) and PFS [41, 42]; this kind of research could help clinicians to optimise the tools and timing (before, during or after FCR completion), to exploit the complex interactions between CLL and normal immune cells.

Our cohort confirmed the high rate of infection, previously observed, during the first two years following FCR (Fig. 5) [17]. It is therefore perhaps not surprising that a low EOT CD4 count was not associated with an increased risk of infection, which means that a CD4 cell count is not useful to manage anti-viral or microbial prophylaxes, in clinical practice (the 200/mm^3^ threshold for discontinuing prophylactic measures was first suggested by HIV-treating physicians, but has never been validated in onco-heamatology patients [22, 23]). Monitoring of NK cells may be more informative to predict possible infectious complications in these patients (we indeed found a trend between low EOT NK cells and infectious events). Some authors have recently suggested a protective role of NK cells in CLL, not in terms of progression of disease, but in terms of OS, corroborating our observation [43]. However, these authors did not study the influence of NK cells on infections, nor the impact of NK cells after frontline CIT. Secondary cancer rates in our cohort were found to be comparable to those reported in the literature [4, 5], but only in terms of Richter transformation: it is noteworthy that our rate of myelodysplastic syndromes/AML was unusually low (2/162) when compared to the MDACC FCR300 cohort (14/300), but our follow-up duration was much shorter. This cannot be explained by dose intensity of FCR, since the French oral FC regimen is slightly over-dosed compared to intravenous FC. In the latter series, 59/300 patients developed solid tumours (28 non-melanoma skin cancers), as compared to 15/162 patients in our cohort. No correlations with EOT lymphocyte counts could be drawn from our analyses.

## Conclusion

Our data suggests that in real-life clinical practice, CD4 cell counts should be assessed after completing FCR, not to stop prophylaxes, but as an opportunity to discuss our patient’s recruitment into a clinical trial assessing maintenance, or to mitigate our multiple concerns about prognostication, response durations and/or infectious risks. This parameter is easily available in most centres, but does not replace MRD as the best post-therapy evaluation tool (it is not the “MRD of the poor”). We think there is a window of opportunity to develop post-FCR T-cell targeted (not only B-cell-targeted with antiCD20 antibodies) strategies aiming at eradicating B/T-cell interactions driving subsequent clinical relapses.

## Data Availability

The data that support the findings of this study are available from the corresponding author upon reasonable request.

## Abbreviations

CIT: chemo-immunotherapy
CLL: chronic lymphocytic leukaemia
CR: complete response
CRi: complete response with incomplete bone marrow recovery
EOT: end of treatment
FCR: fludarabine-cyclophosphamide-rituximab
FU: follow-up
HIV: human immunodeficiency virus
HR: hazard ratio
IGHV-M: mutated IGHV status
IGHV-UM: unmutated IGHV status
IWCLL: international workshop on chronic lymphocytic leukaemia
MRD: minimal residual disease
OS: overall survival
PFS: progression-free survival
PR: partial response
